# Awareness of Common Eye Diseases and Their Risk Factors—A Nationwide Cross-Sectional Survey among Adults in Poland

**DOI:** 10.3390/ijerph20043594

**Published:** 2023-02-17

**Authors:** Agnieszka Kamińska, Jarosław Pinkas, Iwona Wrześniewska-Wal, Janusz Ostrowski, Mateusz Jankowski

**Affiliations:** 1 Faculty of Medicine, Collegium Medicum, Cardinal Stefan Wyszynski University, 01-938 Warsaw, Poland; 2 School of Public Health, Centre of Postgraduate Medical Education, 01-826 Warsaw, Poland

**Keywords:** eye diseases, glaucoma, age-related macular degeneration (AMD), risk factors, prevention, knowledge, awareness, Poland

## Abstract

Public knowledge and awareness of eye diseases may influence individuals’ behaviors toward the use of eye care services and prevention methods. The objective of this study was to assess the awareness of common eye diseases and their risk factors among adults in Poland as well as to identify factors associated with knowledge of eye diseases. This nationwide cross-sectional web-based survey was carried out in December 2022 on a representative sample of 1076 adults in Poland. Most of the respondents had heard of cataracts (83.6%), glaucoma (80.7%), conjunctivitis (74.3%), and hordeolum (73.8%). Awareness of dry eye syndrome was declared by 50% of respondents, and 40% were aware of retinal detachment. Among the respondents, 32.3% had heard of AMD, and 16.4% had heard of diabetic retinopathy. A lack of awareness of glaucoma was declared by 38.1% of respondents, and 54.3% declared a lack of awareness of risk factors for AMD. Gender, age, and the presence of chronic diseases were the most important factors (*p* < 0.05) associated with awareness of common eye diseases and risk factors for glaucoma and AMD. This study demonstrated a low level of awareness of common eye diseases among adults in Poland. Personalized communication on eye diseases is needed.

## 1. Introduction

More than 2 billion people worldwide have vision impairment [1,2]. Refractive errors and age-related eye diseases are the most common causes of vision impairment [1,2,3]. The World Health Organization (WHO) estimates that between 2020 and 2030, the global number of people with glaucoma will increase by 1.3 times (from 76 million to 95.4 million) and the global number of people with age-related macular degeneration (AMD) will increase by 1.2 times (from 195.6 million to 243.3 million) [2]. As a result of an aging population, a rise in the number of people with cataracts (especially those aged 70 years and over) has been projected [2]. Moreover, changes in lifestyle and exposure to environmental factors (e.g., air pollution, low humidity, and winds) may also increase the number of people with eye irritation or dry eye symptoms [4,5].

High public knowledge of common eye diseases is important for reducing the global burden of eye diseases [2,6]. Many eye diseases (e.g., glaucoma) can be asymptomatic/mildly symptomatic for a long time [7]. Older people may misinterpret the reduction in vision as part of the normal aging process instead of an eye disease symptom [8]. Low awareness of eye diseases may lead to delays in seeking medical care and prolong the time from the onset of symptoms to diagnosis [2]. Education on common eye diseases may increase the level of public knowledge on eye diseases and change individual behaviors, which may promote early detection and treatment of eye diseases and encourage people at risk to seek regular eye care [9].

Age is the most important factor for numerous eye diseases [2,10]. Genetic predisposition is also a well-described risk factor for age-related eye diseases [11]. However, there are also modifiable risk factors related to lifestyle behaviors [12,13,14]. Tobacco use, nutrition, and occupational exposure are common lifestyle factors associated with eye disorders and diseases [2,12,13,14]. Smokers are at higher risk of AMD, cataracts, uveitis, and optic neuropathies [14,15]. Smoking during pregnancy also increases the risk of strabismus and optic nerve hypoplasia [14]. Obesity increases the risk of cataracts, AMD, and diabetic retinopathy [16]. Vitamin A (beta-carotene) deficiency can cause corneal opacity and macular degeneration [17]. Excessive sunbath (including UV-B exposure), corticosteroid use, and diabetes are well-known factors for cataracts [18]. Scientific data suggest that arterial hypertension [19] and a cholesterol-enriched diet [20,21] may increase the risk for AMD. Diabetic retinopathy is a serious complication of diabetes that can be prevented/delayed with lifestyle changes and proper control of blood glucose levels [22]. Diabetes is also a risk factor for glaucoma [23]. Awareness of risk factors for eye diseases is necessary to implement eye disease prevention at the individual and population levels.

Public knowledge and awareness of common eye diseases (including cataracts, glaucoma, AMD, and dry eye symptoms) were assessed in several cross-sectional studies around the world [24,25,26,27,28,29,30]. However, most of them are focused on local nonrepresentative populations and were carried out over 5 years ago [24,25,26,27,28]. Most of the studies on public awareness of common eye diseases were carried out in low- or middle-income countries in Asia, and there is a gap in up-to-date nationwide data on awareness of common eye diseases in Europe [24,27,28]. Data on awareness of eye diseases (especially age-related ones) and their risk factors may be used by policymakers to implement eye care programs and preventive strategies [31]. Identification of the factors associated with the level of knowledge on eye diseases and their risk factors may form a basis for educational campaigns and personalized communication.

Therefore, the objective of this study was to assess the awareness of common eye diseases and their risk factors among adults in Poland as well as to identify factors associated with knowledge of eye diseases.

## 2. Materials and Methods

### 2.1. Study Design and Population

This is a nationwide cross-sectional web-based survey. Data were generated as a part of the scientific project entitled “Poles’ attitudes towards eye diseases-knowledge about eye diseases, awareness of risk factors, prevention” [32]. Data were collected between 9 and 12 December 2022 using a dedicated web-based research platform managed by a public opinion research company in Poland [33]. Participants were recruited from over 100,000 adults registered on the research platform [33], and nonprobability quota sampling was used. The stratification model included three variables (gender, age, and place of residence) and was based on the Demographic Yearbook published by Statistics Poland [34]. The sampling methods used in this study allowed us to obtain a nationwide representative sample of adults in Poland [35,36].

Participation was voluntary, and the data collection process was anonymous. Informed consent was obtained. Approval (no. 154/2022) of the Ethical Committee of the Centre of Postgraduate Medical Education in Warsaw was obtained.

### 2.2. Measures

A self-prepared questionnaire was used. During the preparation of the questionnaire, a literature review was performed using the PubMed database [24,25,26,27,28,29,30]. To identify current trends in eye health research, the World Report on Vision published by the WHO was analyzed [2]. The study questionnaire included 9 questions (5 multiple-choice and 4 single-choice) on eye health, preventive behaviors, and knowledge of eye diseases.

*Knowledge of eye diseases*: Respondents were asked to rate their self-reported level of knowledge of eye diseases using a 5-point Likert scale (very bad, rather bad, moderate, rather good, or very good).

*Awareness of common eye diseases*: Respondents were asked about the awareness of common eye diseases using the following question: “Have you ever heard of the eye diseases listed below? (tick all that apply): (1) cataract; (2) glaucoma; (3) age-related macular degeneration (AMD); (4) dry eye syndrome; (5) diabetic retinopathy; (6) diabetic macular edema/diabetic maculopathy (DME); (7) retinal detachment; (8) conjunctivitis; (9) barley on the eye (hordeolum); (10) chalazion”.

*Awareness of risk factors for glaucoma*: Respondents were asked about awareness of risk factors for glaucoma using the following question: “What do you think are the risk factors for glaucoma? (tick all that apply): (1) older age; (2) genetic predisposition (history of glaucoma in the family); (3) tobacco use; (4) excessive alcohol consumption; (5) excessive sunbath; (6) unhealthy diet; (7) arterial hypertension; (8) diabetes; (9) taking selected medications (e.g., steroids); (10) refractive error; or (11) I do not know/none of above”. Based on the literature review [2,15,16,17,18,19,20,21], older age, genetic predisposition, taking selected medications (e.g., steroids), refractive error, arterial hypertension, and diabetes were classified as correct answers.

*Awareness of risk factors for AMD*: Respondents were asked about awareness of risk factors for AMD using the following question: “What do you think are the risk factors for age-related macular degeneration (AMD)? (tick all that apply): (1) older age; (2) genetic predisposition (history of AMD in the family); (3) Caucasian race; (4) tobacco use; (5) excessive alcohol consumption; (6) unhealthy diet; (7) excessive sunbath; (8) arterial hypertension; (9) dyslipidemia; (10) taking selected medications; or (11) I do not know/none of above”. Based on the literature review [2,15,16,17,18,19,20,21], older age, genetic predisposition, Caucasian race, tobacco use, unhealthy diet, and arterial hypertension were classified as correct answers.

*Personal characteristics*: A set of questions on sociodemographic characteristics (gender, age, educational level, marital status, place of residence, having children, occupational activity, and economic status), health status (presence of chronic diseases), and wearing spectacles or contact lenses was addressed.

### 2.3. Statistical Analysis

Data were analyzed with the IBM SPSS software v. 28. Descriptive statistics were used to present categorical data. Cross-tabulation with a chi-squared test was used to compare categorical variables. Multivariable logistic regression analysis was also performed. Demographic data, health status, and wearing spectacles/contact lenses were defined as independent variables. Awareness of eye diseases (10 different models, each disease as a dependent variable) and risk factors for glaucoma (6 different models, each risk factor as a dependent variable) or AMD (6 different models, each risk factor as a dependent variable) was analyzed. The strength of association was presented by the odds ratio (OR) with 95% confidence intervals (95%CI). The statistical significance level was set at *p* < 0.05.

## 3. Results

The study population included 1076 adults; 45.8% were males, 44.4% of respondents had at least one chronic disease, and 55.6% wore spectacles or contact lenses (Table 1).

### 3.1. Awareness of Common Eye Diseases and Their Risk Factors

A very good or rather good level of knowledge of eye diseases was declared by 10% of respondents, and 46.3% declared a rather bad or very bad level of knowledge of eye diseases (Table 2). Among the respondents, 83.6% had heard of cataracts, 80.7% had heard of glaucoma, and 74.3% of respondents had heard of conjunctivitis. Awareness of dry eye syndrome was declared by 50% of respondents, and 40% were aware of retinal detachment. Among the respondents, 32.3% had heard of AMD, and 16.4% had heard of diabetic retinopathy (Table 2).

Older age was the most recognized (39.4%) risk factor for glaucoma. A history of glaucoma in the family was indicated by 27.1% of respondents as a risk factor for glaucoma, while 26% indicated arterial hypertension and 23.2% indicated diabetes. Among the respondents, 38.1% declared a lack of awareness of risk factors for glaucoma, and 10% of respondents incorrectly indicated glaucoma risk factors (Table 2).

A lack of awareness of risk factors for AMD was declared by 54.3% of respondents. Among the respondents, 22.3% correctly indicated that older age is a risk factor for AMD, 13.4% were aware that genetic predisposition is a risk factor for AMD, and 12.9% of respondents indicated arterial hypertension as a risk factor for AMD (Table 2). Details are presented in Table 2.

### 3.2. Sociodemographic Differences in the Awareness of Eye Diseases and Their Risk Factors

There were significant sociodemographic differences in the percentage of respondents who declared awareness of eye diseases (Table 3). Females compared to males were more aware of 9 of 10 eye diseases analyzed in this study. The percentage of respondents who were aware of eye diseases increased with age (*p* < 0.05). Respondents with higher education more often declared that they had heard of common eye diseases listed in this study. The percentage of respondents who had heard of eye diseases was higher among those who had children, those with chronic diseases, and those who wear spectacles or contact lenses (Table 3).

Females, older respondents, and those with chronic diseases more often (*p* < 0.05) declared that older age is a risk factor for glaucoma (Table 4). The percentage of respondents who indicated genetic predisposition as a risk factor for glaucoma significantly differed (*p* < 0.05) by sociodemographic variables (Table 4). Chronically ill respondents more often declared that taking selected medications (e.g., steroids) may increase the risk for glaucoma (*p* < 0.05). Those who were not married more often declared that refractive error may increase the risk for glaucoma (*p* < 0.05). There were also sociodemographic differences in the percentage of respondents who were aware that arterial hypertension or diabetes may increase the risk for glaucoma (Table 4).

The percentage of respondents who declared that genetic predisposition and dyslipidemia are risk factors for AMD was higher (*p* < 0.05) among females than males (Table 5). The percentage of respondents who indicated older age or arterial hypertension as a risk factor for AMD increased with the age (*p* < 0.05). Respondents with a passive occupational status more often declared that older age and genetic predisposition are risk factors for AMD (*p* < 0.05). Those with bad economic status, when compared to those with moderate economic status, more often declared that tobacco use is a risk factor for AMD. Respondents with chronic diseases more often (*p* < 0.05) indicated older age, genetic predisposition, and dyslipidemia as risk factors for AMD. Those who wore spectacles or contact lenses more often indicated older age and genetic predisposition as risk factors for AMD (Table 5).

### 3.3. Factors Associated with Knowledge of Common Eye Diseases

Out of 10 different variables included in multivariable logistic regression analysis (Table 6), gender, age, and the presence of chronic diseases were the most important factors associated with higher awareness of common eye diseases (*p* < 0.05). Wearing spectacles or contact lenses was significantly associated with higher odds of awareness of AMD or dry eye syndrome (*p* < 0.05). Place of residence, having children, and economic status were significantly associated with higher awareness of only 1 of 10 analyzed eye diseases. There was no impact of occupational activity on awareness of common eye diseases (*p* > 0.05). Details are presented in Table 6.

Females were more likely to indicate older age (OR: 1.42, 95%CI: 1.09–1.84, *p* = 0.01), genetic predisposition (OR: 2.47, 95%CI: 1.83–3.34, *p* < 0.001), and arterial hypertension (OR: 1.44, 95%CI: 1.07–1.93, *p* = 0.02) as risk factors for glaucoma (Table 7). Respondents aged 65 years and over were more likely to indicate arterial hypertension (OR: 2.69, 95%CI: 1.60–4.53, *p* < 0.001) and diabetes (OR: 2.02, 95%CI: 1.19–3.44) as risk factors for glaucoma. Respondents aged 50–64 years were more likely to indicate older age as a risk factor for glaucoma (OR: 1.59, 95%CI: 1.09–2.32, *p* = 0.02). Those with higher education were more likely to indicate genetic predisposition as a risk factor for glaucoma (OR: 1.56, 95%CI: 1.16–2.10, *p* = 0.003). Those who were not married were more likely to indicate refractive error as a risk factor for glaucoma (OR: 1.71, 95%CI: 1.03–2.84, *p* = 0.04). Respondents who lived in a city with ≥100,000 and <500,000 inhabitants were more likely to indicate arterial hypertension (OR: 1.61, 95%CI: 1.09–2.39, *p* = 0.02) and taking medication (OR: 1.83, 95%CI: 1.01–3.31, *p* = 0.04) as risk factors for glaucoma (Table 7). Respondents wearing spectacles or contact lenses were more likely to indicate refractive error as a risk factor for glaucoma (OR: 1.67, 95%CI: 1.07–2.61, *p* = 0.02). The presence of chronic diseases was associated with a higher awareness of four different risk factors for glaucoma. There was no impact of occupational activity, economic activity, or having children on awareness of risk factors for glaucoma (Table 7).

Out of 10 different variables analyzed in this study, female gender, older age, and the presence of chronic diseases were significantly associated with awareness of selected risk factors for AMD (Table 8).

## 4. Discussion

To the best of the authors’ knowledge, this is the first nationwide study on awareness of common eye diseases among adults in Poland. Cataracts and glaucoma were the most recognized eye diseases. AMD and diabetic retinopathy—serious eye diseases—were recognized by less than one-third of the participants. A low level of awareness of lifestyle-related risk factors for glaucoma and AMD was observed. Gender, age, and the presence of chronic disease were the most important factors associated with higher awareness of eye diseases and risk factors for glaucoma and AMD. Factors associated with awareness of eye diseases varied depending on the analyzed diseases. However, there was no impact of economic status, occupational status, and place of residence on awareness of eye diseases.

In this study, over 75% of respondents declared awareness of cataracts, glaucoma, conjunctivitis, and hordeolum. Findings from the study (2017) carried out with 802 adults in Jordan showed that dry eye syndrome was the most recognized eye disease (51.9%), followed by glaucoma (38.8%), diabetic retinopathy (37.3%), and cataracts (31.4%) [29]. In a study from Canada (2006), 69.2% declared awareness of cataracts, 41.2% were aware of glaucoma, and 20.2% were aware of macular degeneration [25]. In Iran (2014), 86.2% of adults declared awareness of diabetic retinopathy, 82.9% were aware of cataracts, and 46.6% were aware of glaucoma [37]. In Bangladesh (2015), 90% of adults declared awareness of cataracts, and 86% were aware of trachoma, but only 4% had heard of diabetic retinopathy, 7% were aware of glaucoma, and 8% were aware of AMD [28]. Little is known about awareness of eye diseases among adults in Europe. Findings from this study showed that adults in Poland are more aware of common eye diseases compared to previously published data. However, most of the studies were published over 5 years ago [25,26,27,28,29], so data should be analyzed carefully, and direct comparisons are difficult. We can hypothesize that the level of awareness of eye diseases increases in line with the development of the country as well as the availability of eye care services.

Previously published [24,25,26,27,28,29,30,37] studies showed that female gender, older age, higher education, and good economic status are associated with better knowledge of eye diseases. In this study, females were also more likely to declare awareness of common eye diseases. Those with higher education were more aware of most of the analyzed eye diseases, which is in line with previously published data. However, findings from this study also showed that the presence of chronic diseases is an important factor associated with better knowledge of eye diseases. Contrary to previously published data [28,29], economic status or occupational status did not influence the awareness of eye diseases. Further studies from high-income countries (especially European) are needed.

In this study, factors associated with awareness of risk factors for glaucoma and AMD were also analyzed. Gender, age, and the presence of chronic diseases were the most important factors associated with awareness of risk factors for glaucoma and AMD. We can hypothesize that older adults are more aware of risk factors for these diseases, as age is a risk factor for eye diseases, so they may seek eye care and may be educated by their primary care physicians or ophthalmologists [2,10]. Females more often seek health-related information [38], so we can hypothesize that gender differences in health information behavior may also influence the awareness of eye diseases. Chronic diseases such as diabetes, arterial hypertension, and dyslipidemia are risk factors for eye diseases [2,19,20,21,22], so we can hypothesize that individuals with chronic diseases were informed about eye complications caused by chronic diseases such as diabetes or hypertension such that they are more aware of eye diseases and their risk factors.

AMD is a significant public health problem, and tobacco use is the major risk factor for AMD [14,15]. In this study, less than one-third of adults in Poland were aware of AMD. Findings from this study revealed that more than half of adults in Poland were unaware of any of the risk factors for AMD, and less than one-tenth were aware of the link between smoking and AMD. This study revealed that education on AMD and AMD preventive programs should be a priority for public health authorities in Poland, as significant gaps in public knowledge on AMD were observed.

Individuals who wear spectacles or contact lenses are more likely to use eye care services and visit ophthalmologists/opticians, as they need regular eye examinations and adjustment of spectacles/contact lenses to the refractive error [39]. However, in this study, wearing spectacles or contact lenses was not significantly associated with awareness of most of the analyzed eye diseases. Moreover, there were no significant differences in awareness of risk factors for glaucoma or AMD based on the status of wearing spectacles or contact lenses. This finding suggests that patients did not receive sufficient eye health education when using eye care services.

### 4.1. Practical Implications

This study provided nationwide data on awareness of common eye diseases in Poland. The global burden of eye diseases, especially those that are age-related, will increase, so public health interventions are needed to prepare national health systems for future challenges resulting from the growing incidence of eye diseases. A low level of awareness of eye diseases (especially AMD) underlines an urgent need for education programs on eye health. Most adults in Poland were not aware of modifiable risk factors for AMD (tobacco use and unhealthy diet). Moreover, there were no differences in awareness of risk factors for eye diseases between those who wore spectacles/contact lenses and those without refractive errors, which suggests that ophthalmologists do not pay enough attention to educate patients with refractive errors on eye health and eye diseases. Education on eye health and common eye diseases should be included in the National Health Strategy of the Republic of Poland, as vision is a crucial sense that affects individual social and economic activity. Findings from this study may form a basis for educational campaigns.

### 4.2. Limitations

This study has several limitations typical for cross-sectional studies. Data were self-reported, so recall bias may occur. This analysis was limited to 10 common eye diseases. Questions on diagnosis, symptoms, or treatment methods of common eye diseases were not addressed. The question on the source of knowledge on eye diseases was also missed. Health status and wearing spectacles or contact lenses were self-reported, and medical records were not verified. Nevertheless, this is the first nationwide study on awareness of eye diseases among adults in Poland.

## 5. Conclusions

A low level of awareness of common eye diseases among adults in Poland was observed. Most adults in Poland were not aware of risk factors for age-related eye diseases, such as glaucoma or AMD. Gender, age, and the presence of chronic diseases were the most important factors associated with higher awareness of common eye diseases and risk factors for glaucoma or AMD. A future educational campaign should include these gaps, and personalized communication on eye diseases should be implemented. Findings from this study indicate the need to strengthen eye health knowledge among adults in Poland.

## Figures and Tables

**Table 1:** Characteristics of the study population (n = 1076).

Variable	n	%
Gender		
female	583	54.2
male	493	45.8
Age group (years)		
18–34	337	31.3
35–49	279	25.9
50–64	298	27.7
65+	162	15.1
Educational level		
higher	443	41.2
less than higher	633	58.8
Married		
yes	561	52.1
no	515	47.9
Place of residence		
rural area	403	37.5
city < 20,000 inhabitants	136	12.6
city ≥ 20,000 and <100,000 inhabitants	212	19.7
city ≥ 100,000 and <500,000 inhabitants	191	17.8
city ≥ 500,000 inhabitants	134	12.5
Having children		
yes	688	63.9
no	388	36.1
Occupational activity		
active	653	60.7
passive	423	39.3
Economic status		
good	414	38.5
moderate	408	37.9
bad	254	23.6
Presence of chronic diseases		
yes	478	44.4
no	598	55.6
Wearing spectacles or contact lenses		
yes	598	55.6
no	482	44.8

**Table 2:** Respondents’ knowledge regarding common eye diseases (n = 1076).

Variable	n	%
**Self-reported level of knowledge of eye diseases**		
very good	18	1.7
rather good	89	8.3
moderate	471	43.8
rather bad	360	33.5
very bad	138	12.8
**Have you ever heard of the eye diseases listed below? (tick all you have heard of)**		
cataract	899	83.6
glaucoma	868	80.7
age-related macular degeneration (AMD)	348	32.3
dry eye syndrome	538	50.0
diabetic retinopathy	176	16.4
diabetic macular edema/diabetic maculopathy (DME)	92	8.6
retinal detachment	430	40.0
conjunctivitis	799	74.3
barley on the eye (hordeolum)	794	73.8
chalazion	259	24.1
**What do you think are the risk factors for glaucoma? (tick all that apply)**		
Correct answers (strong or moderate evidence)		
older age	424	39.4
genetic predisposition (history of glaucoma in the family)	292	27.1
taking selected medications (e.g., steroids)	93	8.5
refractive error	105	9.8
arterial hypertension	280	26.0
diabetes	250	23.2
Incorrect answers (lack of evidence or conflicting evidence)		
tobacco use	114	10.6
excessive alcohol consumption	76	7.1
excessive sunbath	62	5.8
unhealthy diet	111	10.3
I do not know/none of above	410	38.1
**What do you think are the risk factors for age-related macular degeneration (AMD)? (tick all that apply)**		
Correct answers (strong or moderate evidence)		
older age	240	22.3
genetic predisposition (history of AMD in the family)	144	13.4
tobacco use	88	8.2
Caucasian race	21	2.0
unhealthy diet	76	7.1
arterial hypertension	139	12.9
dyslipidemia	85	7.9
Incorrect answers (lack of evidence or conflicting evidence)		
excessive alcohol consumption	63	5.9
excessive sunbath	60	5.6
taking selected medications	69	6.4
I do not know/none of above	584	54.3

**Table 3:** Public awareness of selected eye diseases by sociodemographic factors (n = 1076).

	Public Awareness of Selected Eye Diseases									
	Cataract		Glaucoma		AMD		Diabetic Retinopathy		Dry Eye Syndrome	
Variable	%	*p*	%	*p*	%	*p*	%	*p*	%	*p*
Gender										
male	77.7	**<0.001**	74.6	**<0.001**	23.1	**<0.001**	9.1	**<0.001**	35.5	**<0.001**
female	88.5		85.8		40.1		22.5		62.3	
Age group (years)										
18–34	74.5	**<0.001**	71.8	**<0.001**	22.8	**<0.001**	12.2	**0.03**	42.7	**0.01**
35–49	86.0		82.8		29.7		15.4		50.5	
50–64	88.9		87.6		40.6		20.8		56.4	
65+	88.3		82.7		41.4		18.5		52.5	
Educational level										
higher	86.5	**0.03**	84.0	**0.02**	37.9	**0.001**	19.4	**0.02**	55.1	**0.01**
less than higher	81.5		78.4		28.4		14.2		46.4	
Married										
yes	87.2	**<0.001**	84.1	**0.003**	30.7	0.2	16.0	0.8	49.9	0.9
no	79.6		76.9		34.2		16.7		50.1	
Place of residence										
rural area	81.1	0.6	78.9	0.6	28.8	0.1	13.2	0.1	45.7	**0.02**
city < 20,000 inhabitants	84.6		80.9		32.4		16.2		43.4	
city ≥ 20,000 and <100,000 inhabitants	85.4		82.5		30.2		19.8		56.1	
city ≥ 100,000 and <500,000 inhabitants	85.3		79.6		37.7		16.2		51.3	
city ≥ 500,000 inhabitants	84.3		84.3		38.8		20.9		58.2	
Having children										
yes	88.4	**<0.001**	84.2	**<0.001**	34.6	**0.04**	18.5	**0.01**	52.5	**0.03**
no	75.0		74.5		28.4		12.6		45.6	
Occupational activity										
active	82.4	0.2	79.2	0.1	29.6	**0.02**	13.9	**0.01**	47.9	0.1
passive	85.3		83.0		36.6		20.1		53.2	
Economic status										
good	85.0	0.6	83.6	0.1	31.2	0.5	16.9	0.6	51.7	0.7
moderate	82.4		79.4		34.6		15.0		49.0	
bad	83.1		78.0		30.7		17.7		48.8	
Presence of chronic diseases										
yes	90.0	**<0.001**	87.2	**<0.001**	39.5	**<0.001**	24.9	**<0.001**	58.8	**<0.001**
no	78.4		75.4		26.6		9.5		43.0	
Wearing spectacles or contact lenses										
yes	87.0	**<0.001**	83.7	**0.01**	39.1	**<0.001**	19.5	**0.002**	56.7	**<0.001**
no	79.3		77.0		24.1		12.4		41.7	
	**Diabetic** **Macular Edema/** **Diabetic** **Maculopathy (DME)**		**Retinal** **Detachment**		**Conjunctivitis**		**Barley on the Eye** **(Hordeolum)**		**Chalazion**	
**Variable**	**%**	***p***	**%**	***p***	**%**	***p***	**%**	***p***	**%**	***p***
Gender										
male	7.7	0.4	33.1	**<0.001**	66.3	**<0.001**	64.7	**<0.001**	15.6	**<0.001**
female	9.3		45.8		81.0		81.5		31.2	
Age group (years)										
18–34	10.7	0.4	33.5	**0.03**	68.0	**0.004**	62.9	**<0.001**	20.5	0.1
35–49	6.8		44.1		74.9		76.3		22.9	
50–64	8.1		42.6		80.5		81.2		28.2	
65+	8.0		41.4		74.7		78.4		25.9	
Educational level										
higher	9.0	0.6	46.0	**<0.001**	78.6	**0.01**	78.3	**0.01**	28.7	**0.003**
less than higher	8.2		35.7		71.2		70.6		20.9	
Married										
yes	6.6	**0.02**	39.2	0.6	77.0	**0.03**	78.4	**<0.001**	25.0	0.5
no	10.7		40.8		71.3		68.7		23.1	
Place of residence										
rural area	6.5	0.2	36.0	0.1	71.7	0.2	70.5	0.3	17.9	**0.004**
city < 20,000 inhabitants	9.6		44.9		75.7		73.5		25.7	
city ≥ 20,000 and <100,000 inhabitants	11.8		39.2		72.2		75.5		29.2	
city ≥ 100,000 and <500,000 inhabitants	7.3		40.8		75.9		74.9		25.7	
city ≥ 500,000 inhabitants	10.4		47.0		81.3		79.9		30.6	
Having children										
yes	8.4	0.9	43.5	**0.002**	77.5	**0.001**	79.2	**<0.001**	26.0	**0.04**
no	8.8		33.8		68.6		64.2		20.6	
Occupational activity										
active	8.3	0.7	39.7	0.8	73.3	0.9	72.7	0.3	24.3	0.8
passive	9.0		40.4		74.2		75.4		23.6	
Economic status										
good	9.4	0.7	38.6	0.5	77.1	0.3	75.6	0.4	24.6	0.8
moderate	8.3		39.5		72.3		73.8		24.5	
bad	7.5		42.9		72.8		70.9		22.4	
Presence of chronic diseases										
yes	10.7	**0.03**	45.0	**0.003**	79.3	**<0.001**	81.4	**<0.001**	28.9	**<0.001**
no	6.9		36.0		70.2		67.7		20.2	
Wearing spectacles or contact lenses										
yes	9.4	0.3	42.4	0.07	77.8	**0.003**	76.9	**0.01**	27.1	**0.01**
no	7.5		36.9		69.9		69.9		20.3	

All statistically significant variables are bolded.

**Table 4:** Public awareness of risk factors for glaucoma by sociodemographic factors (n = 1076).

Public Awareness of Risk Factors for Glaucoma												
	Older Age		Genetic Predisposition		Taking Selected Medications (e.g., Steroids)		Refractive Error		Arterial Hypertension		Diabetes	
Variable	%	*p*	%	*p*	%	*p*	%	*p*	%	*p*	%	*p*
Gender												
male	34.3	**0.002**	17.6	**<0.001**	7.5	0.2	9.9	0.9	22.3	**0.01**	23.1	0.9
female	43.7		35.2		9.6		9.6		29.2		23.3	
Age group (years)												
18–34	32.3	**0.01**	23.4	**0.04**	10.4	0.1	12.8	0.08	19.3	**<0.001**	18.7	**<0.001**
35–49	39.4		24.4		10.4		10.0		26.2		21.1	
50–64	45.0		31.2		5.7		8.1		24.5		23.5	
65+	43.8		32.1		7.4		6.2		42.6		35.8	
Educational level												
higher	40.6	0.5	31.6	**0.01**	10.2	0.1	8.8	0.4	28.7	0.1	24.4	0.5
less than higher	38.5		24.0		7.6		10.4		24.2		22.4	
Married												
yes	39.2	0.9	27.6	0.7	7.3	0.1	7.7	**0.02**	27.6	0.2	24.2	0.4
no	39.6		26.6		10.1		12.0		24.3		22.1	
Place of residence												
rural area	37.7	0.8	23.8	0.2	6.7	0.05	10.9	0.2	23.1	0.1	21.6	0.4
city < 20,000 inhabitants	37.5		29.4		7.4		9.6		23.5		23.5	
city ≥ 20,000 and <100,000 inhabitants	40.6		25.5		10.8		12.3		25.9		27.8	
city ≥ 100,000 and <500,000 inhabitants	42.9		32.5		13.1		6.3		33.5		20.9	
city ≥ 500,000 inhabitants	39.6		29.9		6.0		7.5		26.9		23.9	
Having children												
yes	41.4	0.07	27.5	0.7	7.7	0.1	8.9	0.2	28.5	**0.01**	23.7	0.6
no	35.8		26.5		10.3		11.3		21.6		22.4	
Occupational activity												
active	37.5	0.1	23.9	**0.003**	9.0	0.6	11.2	0.05	23.1	**0.01**	19.9	**0.001**
passive	42.3		32.2		8.0		7.6		30.5		28.4	
Economic status												
good	39.4	0.4	27.1	0.4	8.2	0.7	9.4	0.9	27.5	0.7	24.2	0.1
moderate	37.3		28.9		9.6		9.6		25.2		20.1	
bad	42.9		24.4		7.9		10.6		24.8		26.8	
Presence of chronic diseases												
yes	47.3	**<0.001**	33.3	**<0.001**	10.7	**0.03**	9.0	0.5	30.5	**0.003**	28.5	**<0.001**
no	33.1		22.2		7.0		10.4		22.4		19.1	
Wearing spectacles or contact lenses												
yes	41.6	0.1	30.3	**0.01**	9.3	0.4	10.8	0.2	29.1	**0.01**	25.6	**0.04**
no	36.7		23.2		7.9		8.5		22.2		20.3	

All statistically significant variables are bolded.

**Table 5:** Public awareness of risk factors for AMD by sociodemographic factors (n = 1076).

	Public Awareness of Risk Factors for AMD											
	Older Age		Genetic Predisposition		Tobacco Use		Unhealthy Diet		Arterial Hypertension		Dyslipidemia	
Variable	%	*p*	%	*p*	%	*p*	%	*p*	%	*p*	%	*p*
Gender												
male	19.7	0.06	9.7	**0.001**	7.7	0.6	7.1	0.9	13.4	0.7	5.3	**0.003**
female	24.5		16.5		8.6		7.0		12.5		10.1	
Age group (years)												
18–34	16.0	**<0.001**	10.1	0.1	8.6	0.6	7.1	0.9	7.1	**<0.001**	7.1	0.5
35–49	21.1		15.1		9.3		7.2		14.0		6.8	
50–64	25.8		13.1		8.1		6.4		15.4		9.7	
65+	30.9		17.9		5.6		8.0		18.5		8.0	
Educational level												
higher	23.9	0.3	15.8	0.05	8.8	0.5	8.6	0.1	14.7	0.2	9.5	0.1
less than higher	21.2		11.7		7.7		6.0		11.7		6.8	
Married												
yes	22.1	0.9	14.8	0.2	7.5	0.4	7.0	0.9	13.5	0.5	7.7	0.8
no	22.5		11.8		8.9		7.2		12.2		8.2	
Place of residence												
rural area	19.1	0.06	11.9	0.2	6.2	0.1	6.2	0.4	11.2	0.2	7.9	0.5
city < 20,000 inhabitants	17.6		9.6		14.0		10.3		12.5		5.1	
city ≥ 20,000 and <100,000 inhabitants	24.1		16.5		8.5		5.7		15.6		8.0	
city ≥ 100,000 and <500,000 inhabitants	26.2		16.8		7.9		8.4		10.5		10.5	
city ≥ 500,000 inhabitants	28.4		11.9		8.2		6.7		17.9		6.7	
Having children												
yes	23.8	0.1	14.8	0.06	7.8	0.6	6.8	0.7	14.1	0.1	8.1	0.7
no	19.6		10.8		8.8		7.5		10.8		7.5	
Occupational activity												
active	19.1	**0.002**	11.6	**0.04**	7.8	0.6	7.5	0.5	12.4	0.5	6.7	0.1
passive	27.2		16.1		8.7		6.4		13.7		9.7	
Economic status												
good	23.9	0.2	14.5	0.7	8.5	**0.04**	7.7	0.8	12.3	0.7	8.9	0.6
moderate	19.4		12.5		5.9		6.9		14.0		7.1	
bad	24.4		13.0		11.4		6.3		12.2		7.5	
Presence of chronic diseases												
yes	27.6	**<0.001**	17.2	**0.001**	9.8	0.08	7.9	0.3	14.0	0.3	10.3	**0.01**
no	18.1		10.4		6.9		6.4		12.0		6.0	
Wearing spectacles or contact lenses												
yes	25.6	**0.004**	15.3	**0.04**	8.4	0.8	6.9	0.8	14.0	0.3	8.4	0.5
no	18.3		11.0		7.9		7.3		11.6		7.3	

All statistically significant variables are bolded.

**Table 6:** Factors associated with awareness of common eye diseases among adults in Poland (n = 1076).

Factors Associated with Awareness of Common Eye Diseases among Adults in Poland										
	Cataract		Glaucoma		Age-Related Macular Degeneration (AMD)		Diabetic Retinopathy		Dry Eye Syndrome	
Variable	OR (95%CI)	*p*	OR (95%CI)	*p*	OR (95%CI)	*p*	OR (95%CI)	*p*	OR (95%CI)	*p*
Gender										
male	1.00		1.00		1.00		1.00		1.00	
female	2.04 (1.43–2.91)	**<0.001**	1.94 (1.39–2.70)	**<0.001**	2.10 (1.58–2.79)	**<0.001**	2.60 (1.77–3.80)	**<0.001**	2.96 (2.27–3.85)	**<0.001**
Age group (years)										
18–34	1.00		1.00		1.00		1.00		1.00	
35–49	1.72 (1.08–2.73)	**0.02**	1.80 (1.16–2.79)	**0.01**	1.61 (1.07–2.42)	**0.02**	1.24 (0.74–2.08)	0.4	1.49 (1.03–2.16)	**0.03**
50–64	1.74 (1.04–2.91)	**0.04**	2.19 (1.35–3.58)	**0.002**	2.28 (1.51–3.45)	**<0.001**	1.38 (0.82–2.32)	0.2	1.52 (1.04–2.24)	**0.03**
65+	1.52 (0.78–2.97)	0.2	1.29 (0.70–2.35)	0.4	2.17 (1.30–3.63)	**0.003**	0.89 (0.47–1.70)	0.7	1.24 (0.76–2.01)	0.4
Educational level										
higher	1.35 (0.93–1.94)	0.1	1.41 (0.99–1.98)	0.05	1.61 (1.21–2.13)	**0.001**	1.53 (1.07–2.19)	**0.02**	1.37 (1.05–1.80)	**0.02**
less than higher	1.00		1.00		1.00		1.00		1.00	
Married										
yes	1.00	0.9	1.00	0.6	1.00	**0.01**	1.00	**0.04**	1.00	0.2
no	1.03 (0.67–1.58)		0.90 (0.60–1.33)		1.59 (1.15–2.20)		1.51 (1.01–2.25)		1.25 (0.92–1.71)	
Place of residence										
rural area	1.00		1.00		1.00		1.00		1.00	
city < 20,000 inhabitants	1.20 (0.69–2.09)	0.5	1.06 (0.63–1.76)	0.8	1.08 (0.69–1.67)	0.7	1.17 (0.66–2.06)	0.6	0.82 (0.54–1.25)	0.4
city ≥ 20,000 and <100,000 inhabitants	1.29 (0.80–2.08)	0.3	1.19 (0.76–1.87)	0.4	0.91 (0.62–1.34)	0.6	1.41 (0.88–2.26)	0.2	1.43 (1.00–2.05)	0.05
city ≥ 100,000 and <500,000 inhabitants	1.22 (0.74–2.00)	0.4	0.95 (0.60–1.48)	0.8	1.28 (0.87–1.88)	0.2	1.15 (0.69–1.91)	0.6	1.10 (0.76–1.59)	0.6
city ≥ 500,000 inhabitants	1.13 (0.64–1.98)	0.7	1.29 (0.74–2.25)	0.4	1.27 (0.82–1.98)	0.3	1.48 (0.85–2.57)	0.2	1.44 (0.94–2.22)	0.1
Having children										
yes	1.75 (1.11–2.75)	**0.02**	1.14 (0.74–1.74)	0.6	1.09 (0.75–1.58)	0.6	1.57 (0.98–2.54)	0.06	1.08 (0.76–1.53)	0.7
no	1.00		1.00		1.00		1.00		1.00	
Occupational activity										
active	1.00		1.00		1.00		1.00		1.00	
passive	0.91 (0.61–1.38)	0.7	1.11 (0.75–1.64)	0.6	1.05 (0.76–1.45)	0.8	1.31 (0.88–1.97)	0.2	0.99 (0.73–1.35)	0.9
Economic status										
good	1.34 (0.85–2.10)	0.2	1.68 (1.10–2.56)	**0.02**	1.21 (0.84–1.75)	0.3	1.22 (0.78–1.92)	0.4	1.30 (0.92–1.83)	0.1
moderate	0.99 (0.64–1.53)	0.9	1.14 (0.76–1.70)	0.5	1.25 (0.88–1.79)	0.2	0.84 (0.53–1.31)	0.4	1.05 (0.75–1.47)	0.8
bad	1.00		1.00		1.00		1.00		1.00	
Presence of chronic diseases										
yes	1.96 (1.33–2.89)	**<0.001**	1.88 (1.32–2.68)	**<0.001**	1.47 (1.11–1.96)	**0.01**	2.80 (1.94–4.04)	**<0.001**	1.68 (1.28–2.21)	**<0.001**
no	1.00		1.00		1.00		1.00		1.00	
Wearing spectacles or contact lenses										
yes	1.25 (0.87–1.79)	0.2	1.10 (0.79–1.54)	0.6	1.53 (1.15–2.04)	**0.004**	1.27 (0.88–1.84)	0.2	1.46 (1.12–1.92)	**0.01**
no	1.00		1.00		1.00		1.00		1.00	
	**Diabetic Macular Edema/Diabetic Maculopathy (DME)**		**Retinal Detachment**		**Conjunctivitis**		**Barley on the Eye** **(Hordeolum)**		**Chalazion**	
**Variable**	**OR (95%CI)**	***p***	**OR (95%CI)**	***p***	**OR (95%CI)**	***p***	**OR (95%CI)**	***p***	**OR (95%CI)**	**Variable**
Gender										
male	1.00		1.00		1.00		1.00		1.00	
female	1.07 (0.68–1.68)	0.8	1.63 (1.25–2.12)	**<0.001**	2.16 (1.61–2.90)	**<0.001**	2.40 (1.78–3.25)	**<0.001**	2.48 (1.82–3.40)	**<0.001**
Age group (years)										
18–34	1.00		1.00		1.00		1.00		1.00	
35–49	0.59 (0.31–1.11)	0.1	1.42 (0.99–2.04)	0.06	1.29 (0.87–1.92)	0.2	1.66 (1.12–2.48)	**0.01**	1.09 (0.71–1.67)	0.7
50–64	0.60 (0.31–1.15)	0.1	1.25 (0.85–1.83)	0.3	1.63 (1.05–2.52)	**0.03**	1.93 (1.25–2.99)	**0.003**	1.35 (0.88–2.10)	0.2
65+	0.46 (0.20–1.07)	0.1	1.09 (0.67–1.77)	0.7	1.20 (0.70–2.05)	0.5	1.60 (0.92–2.77)	0.1	1.32 (0.76–2.30)	0.3
Educational level										
higher	1.05 (0.66–1.66)	0.9	1.55 (1.19–2.02)	**0.001**	1.38 (1.02–1.88)	**0.04**	1.44 (1.06–1.96)	**0.02**	1.44 (1.06–1.94)	**0.02**
less than higher	1.00		1.00		1.00		1.00		1.00	
Married										
yes	1.00		1.00		1.00		1.00		1.00	
no	2.14 (1.26–3.62)	**0.01**	1.50 (1.10–2.03)	**0.01**	0.96 (0.67–1.36)	0.8	0.92 (0.64–1.31)	0.6	1.03 (0.73–1.45)	0.9
Place of residence										
rural area	1.00		1.00		1.00		1.00		1.00	
city < 20,000 inhabitants	1.47 (0.72–2.98)	0.3	1.42 (0.95–2.13)	0.1	1.19 (0.75–1.89)	0.4	1.08 (0.68–1.71)	0.8	1.53 (0.95–2.46)	0.1
city ≥ 20,000 and <100,000 inhabitants	1.79 (0.99–3.22)	0.05	1.05 (0.74–1.50)	0.8	0.95 (0.65–1.40)	0.8	1.22 (0.81–1.82)	0.3	1.77 (1.18–2.65)	**0.01**
city ≥ 100,000 and <500,000 inhabitants	1.01 (0.51–2.02)	0.9	1.12 (0.78–1.61)	0.6	1.13 (0.75–1.71)	0.6	1.14 (0.75–1.73)	0.5	1.44 (0.94–2.21)	0.1
city ≥ 500,000 inhabitants	1.49 (0.73–3.04)	0.3	1.45 (0.96–2.20)	0.1	1.57 (0.94–2.51)	0.1	1.53 (0.92–2.54)	0.1	1.80 (1.12–2.88)	**0.02**
Having children										
yes	1.74 (0.96–3.17)	0.07	1.65 (1.17–2.34)	**0.01**	1.21 (0.83–1.78)	0.3	1.42 (0.97–2.09)	0.1	1.16 (0.78–1.73)	0.5
no	1.00		1.00		1.00		1.00		1.00	
Occupational activity										
active	1.00		1.00		1.00		1.00		1.00	
passive	1.12 (0.66–1.87)	0.7	0.96 (0.70–1.30)	0.8	0.84 (0.59–1.18)	0.3	0.87 (0.61–1.24)	0.4	0.77 (0.54–1.10)	0.1
Economic status										
good	1.48 (0.81–2.70)	0.2	0.88 (0.63–1.23)	0.5	1.32 (0.90–1.92)	0.2	1.45 (0.99–2.11)	0.07	1.17 (0.79–1.73)	0.4
moderate	1.20 (0.66–2.18)	0.6	0.85 (0.61–1.18)	0.3	0.95 (0.66–1.37)	0.8	1.19 (0.82–1.72)	0.4	1.07 (0.73–1.58)	0.7
bad	1.00		1.00		1.00		1.00		1.00	
Presence of chronic diseases										
yes	1.74 (1.10–2.77)	**0.02**	1.32 (1.01–1.73)	**0.04**	1.41 (1.03–1.92)	**0.03**	1.74 (1.27–2.38)	**<0.001**	1.40 (1.03–1.89)	**0.03**
no	1.00		1.00		1.00		1.00		1.00	
Wearing spectacles or contact lenses										
yes	1.29 (0.81–2.06)	0.3	1.08 (0.82–1.40)	0.6	1.16 (0.86–1.57)	0.3	1.01 (0.74–1.36)	0.9	1.15 (0.85–1.57)	0.4
no	1.00		1.00		1.00		1.00		1.00	

All statistically significant variables are bolded.

**Table 7:** Factors associated with awareness of risk factors for glaucoma (n = 1076).

Factors Associated with Awareness of Risk Factors for Glaucoma												
	Older Age		Genetic Predisposition (History of Glaucoma in the Family)		Taking Selected Medications (e.g., Steroids)		Refractive Error		Arterial Hypertension		Diabetes	
Variable	OR (95%CI)	*p*	OR (95%CI)	*p*	OR (95%CI)	*p*	OR (95%CI)	*p*	OR (95%CI)	*p*	OR (95%CI)	*p*
Gender												
male	1.00		1.00		1.00		1.00		1.00		1.00	
female	1.42 (1.09–1.84)	**0.01**	2.47 (1.83–3.34)	**<0.001**	1.25 (0.79–1.98)	0.3	0.98 (0.64–1.50)	0.9	1.44 (1.07–1.93)	**0.02**	0.97 (0.72–1.31)	0.8
Age group (years)												
18–34	1.00		1.00		1.00		1.00		1.00		1.00	
35–49	1.39 (0.97–2.01)	0.07	1.20 (0.79–1.82)	0.4	1.11 (0.62–1.99)	0.7	0.78 (0.45–1.36)	0.4	1.46 (0.97–2.22)	0.07	1.24 (0.81–1.91)	0.3
50–64	1.59 (1.09–2.32)	**0.02**	1.49 (0.98–2.28)	0.06	0.50 (0.25–1.01)	0.05	0.55 (0.30–1.02)	0.06	1.20 (0.77–1.86)	0.4	1.28 (0.81–2.00)	0.3
65+	1.42 (0.88–2.29)	0.2	1.39 (0.82–2.35)	0.2	0.64 (0.27–1.49)	0.3	0.50 (0.21–1.19)	0.1	2.69 (1.60–4.53)	**<0.001**	2.02 (1.19–3.44)	**0.01**
Educational level												
higher	1.10 (0.84–1.43)	0.5	1.56 (1.16–2.10)	**0.003**	1.23 (0.78–1.93)	0.4	0.79 (0.51–1.22)	0.3	1.20 (0.89–1.61)	0.2	1.13 (0.83–1.53)	0.4
less than higher	1.00		1.00		1.00		1.00		1.00		1.00	
Married												
yes	1.00		1.00		1.00		1.00		1.00		1.00	
no	1.25 (0.92–1.69)	0.2	0.92 (0.66–1.29)	0.6	1.29 (0.76–2.18)	0.4	1.71 (1.03–2.84)	**0.04**	0.97 (0.69–1.35)	0.9	0.90 (0.63–1.27)	0.5
Place of residence												
rural area	1.00		1.00		1.00		1.00		1.00		1.00	
city < 20,000 inhabitants	0.94 (0.62–1.41)	0.8	1.21 (0.77–1.89)	0.4	1.01 (0.47–2.17)	0.9	0.91 (0.47–1.76)	0.2	0.95 (0.59–1.52)	0.8	1.07 (0.67–1.71)	0.8
city ≥ 20,000 and <100,000 inhabitants	1.06 (0.75–1.50)	0.8	0.97 (0.65–1.45)	0.9	1.54 (0.85–2.79)	0.2	1.13 (0.67–1.91)	**0.04**	1.07 (0.72–1.59)	0.8	1.33 (0.90–1.97)	0.2
city ≥ 100,000 and <500,000 inhabitants	1.18 (0.82–1.70)	0.4	1.39 (0.94–2.08)	0.1	1.83 (1.01–3.31)	**0.04**	0.50 (0.25–0.98)	0.7	1.61 (1.09–2.39)	**0.02**	0.93 (0.60–1.44)	0.7
city ≥ 500,000 inhabitants	0.99 (0.65–1.51)	0.9	1.14 (0.71–1.81)	0.6	0.68 (0.29–1.58)	0.4	0.62 (0.30–1.29)	0.8	1.09 (0.68–1.75)	0.7	1.04 (0.64–1.68)	0.9
Having children												
yes	1.09 (0.78–1.54)	0.6	0.75 (0.51–1.10)	0.1	0.87 (0.49–1.56)	0.6	1.24 (0.71–2.15)	0.5	1.06 (0.72–1.56)	0.8	0.78 (0.52–1.15)	0.2
no	1.00		1.00		1.00		1.00		1.00		1.00	
Occupational activity												
active	1.00		1.00		1.00		1.00		1.00		1.00	
passive	1.00 (0.74–1.35)	0.9	1.30 (0.93–1.81)	0.1	0.93 (0.54–1.59)	0.8	0.65 (0.39–1.08)	0.1	1.05 (0.75–1.49)	0.8	1.24 (0.88–1.77)	0.2
Economic status												
good	1.00 (0.72–1.40)	0.9	1.28 (0.87–1.88)	0.2	1.06 (0.58–1.93)	0.8	0.84 (0.49–1.44)	0.5	1.25 (0.86–1.82)	0.2	0.95 (0.65–1.38)	0.8
moderate	0.82 (0.59–1.15)	0.2	1.28 (0.88–1.86)	0.2	1.28 (0.72–2.28)	0.4	0.93 (0.54–1.56)	0.8	1.04 (0.71–1.51)	0.9	0.71 (0.49–1.04)	0.08
bad	1.00		1.00		1.00		1.00		1.00		1.00	
Presence of chronic diseases												
yes	1.67 (1.28–2.18)	**<0.001**	1.48 (1.10–1.98)	**0.01**	1.82 (1.15–2.87)	**0.01**	1.04 (0.67–1.62)	0.9	1.28 (0.95–1.72)	0.1	1.44 (1.06–1.95)	**0.02**
no	1.00		1.00		1.00		1.00		1.00		1.00	
Wearing spectacles or contact lenses												
yes	1.01 (0.78–1.32)	0.9	1.12 (0.83–1.51)	0.5	1.26 (0.79–1.99)	0.3	1.67 (1.07–2.61)	**0.02**	1.19 (0.89–1.61)	0.2	1.17 (0.86–1.59)	0.3
no	1.00		1.00		1.00		1.00		1.00		1.00	

All statistically significant variables are bolded.

**Table 8:** Factors associated with awareness of risk factors for AMD (n = 1076).

Factors Associated with Awareness of Risk Factors for AMD												
	Older Age		Genetic Predisposition		Tobacco Use		Unhealthy Diet		Arterial Hypertension		Dyslipidemia	
Variable	OR (95%CI)	*p*	OR (95%CI)	*p*	OR (95%CI)	*p*	OR (95%CI)	*p*	OR (95%CI)	*p*	OR (95%CI)	*p*
Gender												
male	1.00		1.00		1.00		1.00		1.00		1.00	
female	1.22 (0.89–1.65)	0.2	1.77 (1.20–2.60)	**0.004**	1.02 (0.64–1.62)	0.9	1.05 (0.65–1.71)	0.8	0.91 (0.63–1.33)	0.6	1.89 (1.16–3.11)	**0.01**
Age group (years)												
18–34	1.00		1.00		1.00		1.00		1.00		1.00	
35–49	1.50 (0.96–2.35)	0.1	1.53 (0.90–2.60)	0.1	1.13 (0.62–2.09)	0.7	1.04 (0.53–2.04)	0.9	2.20 (1.24–3.92)	**0.01**	1.04 (0.53–2.07)	0.9
50–64	1.69 (1.06–2.67)	**0.03**	1.05 (0.59–1.87)	0.9	0.84 (0.43–1.64)	0.6	1.00 (0.49–2.07)	0.9	2.64 (1.45–4.78)	**0.001**	1.40 (0.71–2.76)	0.3
65+	1.75 (1.01–3.04)	**0.048**	1.35 (0.69–2.64)	0.4	0.44 (0.18–1.09)	0.08	1.41 (0.58–3.45)	0.5	3.23 (1.58–6.59)	**0.001**	0.91 (0.38–2.17)	0.8
Educational level												
higher	1.14 (0.84–1.56)	0.4	1.38 (0.95–2.01)	0.1	1.18 (0.74–1.89)	0.5	1.41 (0.86–2.30)	0.2	1.27 (0.87–1.86)	0.2	1.57 (0.98–2.51)	0.06
less than higher	1.00		1.00		1.00		1.00		1.00		1.00	
Married												
yes	1.00		1.00		1.00		1.00		1.00		1.00	
no	1.26 (0.89–1.78)	0.2	0.87 (0.57–1.34)	0.5	1.24 (0.72–2.13)	0.4	1.03 (0.58–1.82)	0.9	1.11 (0.73–1.71)	0.6	1.23 (0.72–2.10)	0.5
Place of residence												
rural area	1.00		1.00		1.00		1.00		1.00		1.00	
city < 20,000 inhabitants	0.87 (0.52–1.46)	0.6	0.73 (0.38–1.40)	0.3	2.60 (1.37–4.95)	**0.004**	1.64 (0.82–3.28)	0.2	1.09 (0.60–2.00)	0.8	0.58 (0.25–1.36)	0.2
city ≥ 20,000 and <100,000 inhabitants	1.26 (0.83–1.90)	0.3	1.33 (0.82–2.16)	0.2	1.40 (0.74–2.66)	0.3	0.84 (0.41–1.72)	0.6	1.40 (0.86–2.30)	0.2	0.91 (0.48–1.70)	0.8
city ≥ 100,000 and <500,000 inhabitants	1.46 (0.96–2.23)	0.08	1.42 (0.86–2.35)	0.2	1.31 (0.66–2.58)	0.4	1.25 (0.64–2.43)	0.5	0.88 (0.50–1.55)	0.7	1.23 (0.67–2.26)	0.5
city ≥ 500,000 inhabitants	1.58 (0.99–2.54)	0.06	0.89 (0.47–1.66)	0.7	1.33 (0.62–2.85)	0.5	0.91 (0.40–2.05)	0.8	1.62 (0.92–2.85)	0.1	0.71 (0.32–1.58)	0.4
Having children												
yes	1.09 (0.73–1.63)	0.7	1.07 (0.65–1.76)	0.8	1.08 (0.79–2.24)	0.8	0.87 (0.46–1.65)	0.7	0.97 (0.59–1.59)	0.9	1.01 (0.54–1.89)	0.9
no	1.00		1.00		1.00		1.00		1.00		1.00	
Occupational activity												
active	1.00		1.00		1.00		1.00		1.00		1.00	
passive	1.35 (0.95–1.92)	0.1	1.31 (0.85–2.02)	0.2	1.33 (0.79–2.24)	0.3	0.76 (0.42–1.39)	0.4	0.93 (0.60–1.47)	0.8	1.43 (0.85–2.41)	0.2
Economic status												
good	1.13 (0.77–1.66)	0.5	1.24 (0.77–2.00)	0.4	0.75 (0.43–1.28)	0.3	1.21 (0.64–2.30)	0.6	1.05 (0.64–1.72)	0.9	1.41 (0.77–2.59)	0.3
moderate	0.77 (0.52–1.13)	0.2	0.97 (0.60–1.57)	0.9	0.48 (0.27–0.85)	**0.01**	1.06 (0.56–2.02)	0.9	1.18 (0.73–1.90)	0.5	1.00 (0.54–1.85)	0.9
bad	1.00		1.00		1.00		1.00		1.00		1.00	
Presence of chronic diseases												
yes	1.44 (1.06–1.96)	**0.02**	1.59 (1.09–2.31)	**0.02**	1.49 (0.93–2.38)	0.1	1.33 (0.81–2.19)	0.3	0.98 (0.67–1.44)	0.9	1.70 (1.06–2.75)	**0.03**
no	1.00		1.00		1.00		1.00		1.00		1.00	
Wearing spectacles or contact lenses												
yes	1.24 (0.90–1.70)	0.2	1.23 (0.84–1.81)	0.3	1.10 (0.69–1.77)	0.7	0.89 (0.54–1.46)	0.6	1.03 (0.70–1.52)	0.9	0.92 (0.57–1.48)	0.7
no	1.00		1.00		1.00		1.00		1.00		1.00	

All statistically significant variables are bolded.

## Data Availability

Data are available on reasonable request.
